# Cathodal tDCS exerts neuroprotective effect in rat brain after acute ischemic stroke

**DOI:** 10.1186/s12868-020-00570-8

**Published:** 2020-05-12

**Authors:** Ke-Ying Zhang, Gang Rui, Jun-Ping Zhang, Ling Guo, Guang-Zhou An, Jia-Jin Lin, Wei He, Gui-Rong Ding

**Affiliations:** 1 Department of Radiation Biology, Faculty of Preventive Medicine, Fourth Military Medical University, Xi′an, 710032 China; 2 Ministry of Education Key Lab of Hazard Assessment and Control in Special Operational Environment, 169# Chang Le West Road, Xi’an, 710032 China

**Keywords:** tDCS, Stroke, Neuroinflammation, Neuroprotection, Rat

## Abstract

**Background:**

Transcranial direct current stimulation (tDCS) is a non-invasive brain modulation technique that has been proved to exert beneficial effects in the acute phase of stroke. To explore the underlying mechanism, we investigated the neuroprotective effects of cathodal tDCS on brain injury caused by middle cerebral artery occlusion (MCAO).

**Results:**

We established the MCAO model and sham MCAO model with an epicranial electrode implanted adult male Sprague–Dawley rats, and then they were randomly divided into four groups (MCAO + tDCS, MCAO + sham tDCS (Sham), Control + tDCS and Control + Sham group). In this study, the severity degree of neurological deficit, the morphology of brain damage, the apoptosis, the level of neuron-specific enolase and inflammatory factors, the activation of glial cells was detected. The results showed that cathodal tDCS significantly improved the level of neurological deficit and the brain morphology, reduced the brain damage area and apoptotic index, and increased the number of Nissl body in MCAO rats, compared with MCAO + Sham group. Meanwhile, the high level of NSE, inflammatory factors, Caspase 3 and Bax/Bcl2 ratio in MCAO rats was reduced by cathodal tDCS. Additionally, cathodal tDCS inhibited the activation of astrocyte and microglia induced by MCAO. No difference was found in two Control groups.

**Conclusion:**

Our results suggested that cathodal tDCS could accelerate the recovery of neurologic deficit and brain damage caused by MCAO. The inhibition of neuroinflammation and apoptosis resulted from cathodal tDCS may be involved in the neuroprotective process.

## Background

Ischemic stroke, a severe disease caused by cerebral arterial occlusion, can induce multiple pathophysiological processes such as hypoxia of cell, loss of cell homeostasis and acidosis [1]. It was reported that neuronal death could occur within minutes to hours after ischemic stroke, mainly via apoptosis or necrosis [2]. Although the administration of recombinant tissue plasminogen activator and endovascular intervention could restore blood flow perfusion, the ischemia–reperfusion injury may decline the activity of neurons and even result in more serious cell damage [3]. Therefore, establishing an effective way to rescue the neurons suffering from ischemic injury is quite essential for stroke therapy.

Cerebral ischemic penumbra (CIP) is regarded as a vital area of brain injury treatment [4]. The neurons in CIP could be reversibly damaged when the ischemic stroke occurs, and then, neuroinflammation induced by the ischemic injury may aggravate the degree of brain injury and push those endangered neurons into irreversible damage [5]. As reported, microcirculation treatment, hypothermia brain protection, cerebral edema dehydration, and neurotrophic factors injection could exert a neuroprotective effect to some extent [6].

Transcranial direct current stimulation (tDCS), a novel non-invasive brain stimulation, is increasingly investigated in basic and clinical research as an adjuvant tool to promote disease rehabilitation, including ischemic stroke. As reported [7], tDCS performed over M1 area ipsilateral to ischemic stroke significantly improved the patients’ motor function through alternating cortical excitability. Additionally, tDCS was verified to affect non-neuronal tissues such as endothelial cells, lymphocytes, and glial cells, which were supposed to be involved in the process of neuroinflammation [8–11]. However, until now, whether tDCS has the capacity of neuroprotection remains unclear.

In order to clarify the neuroprotective effect of cathodal tDCS on ischemic stroke and explore the underlying mechanism, we performed the present study with the middle cerebral artery occlusion (MCAO) model rats.

## Results

### tDCS promoted the recovery of neurological deficit induced by MCAO operation

**Fig. 1:**
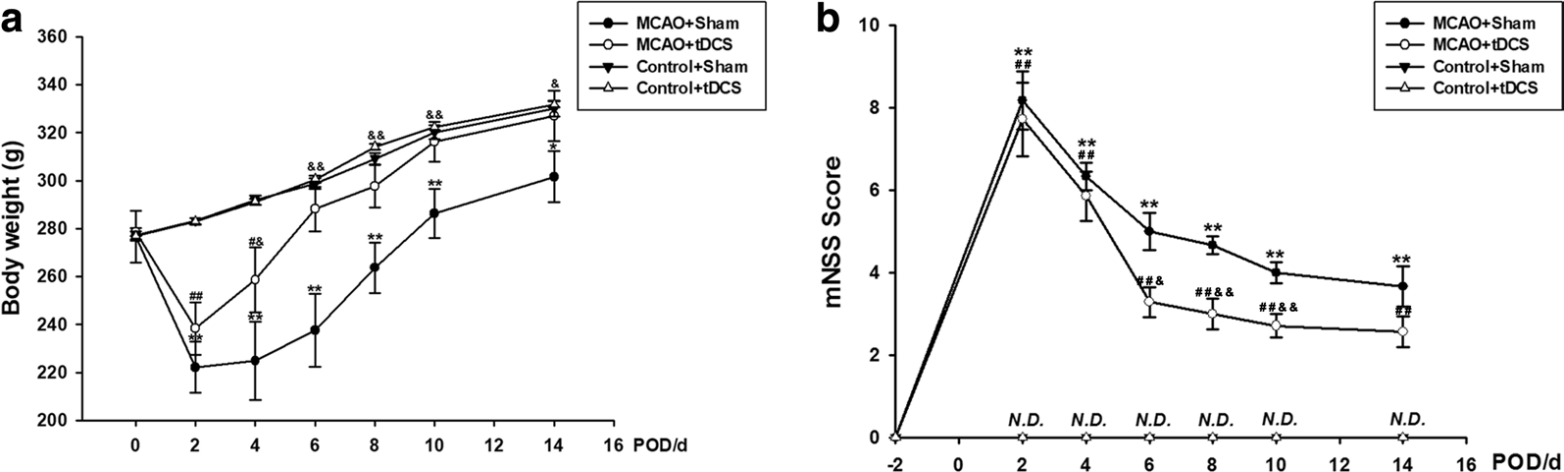
tDCS promoted the recovery of neurological deficit induced by MCAO operation. **a** Body weight monitoring; **b** mNSS result. Data are presented as mean ± standard error of mean (SEM). ^**^P < 0.01 and ^*^P < 0.05, MCAO + Sham *vs* Control + Sham; ^##^*P* < 0.01, MCAO + tDCS *vs* Control + tDCS; ^&&^*P *< 0.01 and ^&^*P* < 0.05, MCAO + Sham *vs* MCAO + tDCS. n = 6 per group for two control groups and MCAO + Sham group. n = 7 for MCAO + tDCS group

As shown in Fig. 1a and Additional file 1: Table S1, there was no obvious difference in body weight before MCAO operation among four groups (*F* = 0.083, *P* = 0.968). Compared with the two control groups, the body weight of MCAO rats significantly decreased on the second day post MCAO operation (POD 2) (*t* = 5.271, *P* = 0.000, MCAO + sham tDCS (Sham) vs Control + Sham; *t* = 4.010, *P* = 0.001, MCAO + tDCS vs Control + tDCS). After 5-day tDCS treatment, the body weight in MCAO + tDCS group significantly increased, compared with MCAO + Sham group (*t* = 3.988, *P* = 0.001). No differences were found in body weight between Control + Sham group and Control + tDCS group (*F* = 0.393, *P* = 0.545).

Modified neurological deficit score (mNSS) is a widely used method for neurological deficit evaluation. As shown in Fig. 1b and Additional file 2: Table S2, mNSS in both control groups was negative throughout the whole experiment, and mNSS in MCAO groups significantly increased after MCAO operation on POD 2 (*t* = 8.462, *P* = 0.000, MCAO + Sham vs Control + Sham; *t* = 8.263, *P* = 0.000, MCAO + tDCS vs Control + tDCS), which indicated that MCAO induced neurological deficit. After tDCS treatment, the mNSS significantly decreased in MCAO + tDCS group, compared with MCAO + Sham group (*F* = 6.528, *P* = 0.027), which suggested that tDCS could significantly promote the recovery of neurological deficit of MCAO rats.

### tDCS alleviated the brain damage induced by MCAO operation

**Fig. 2:**
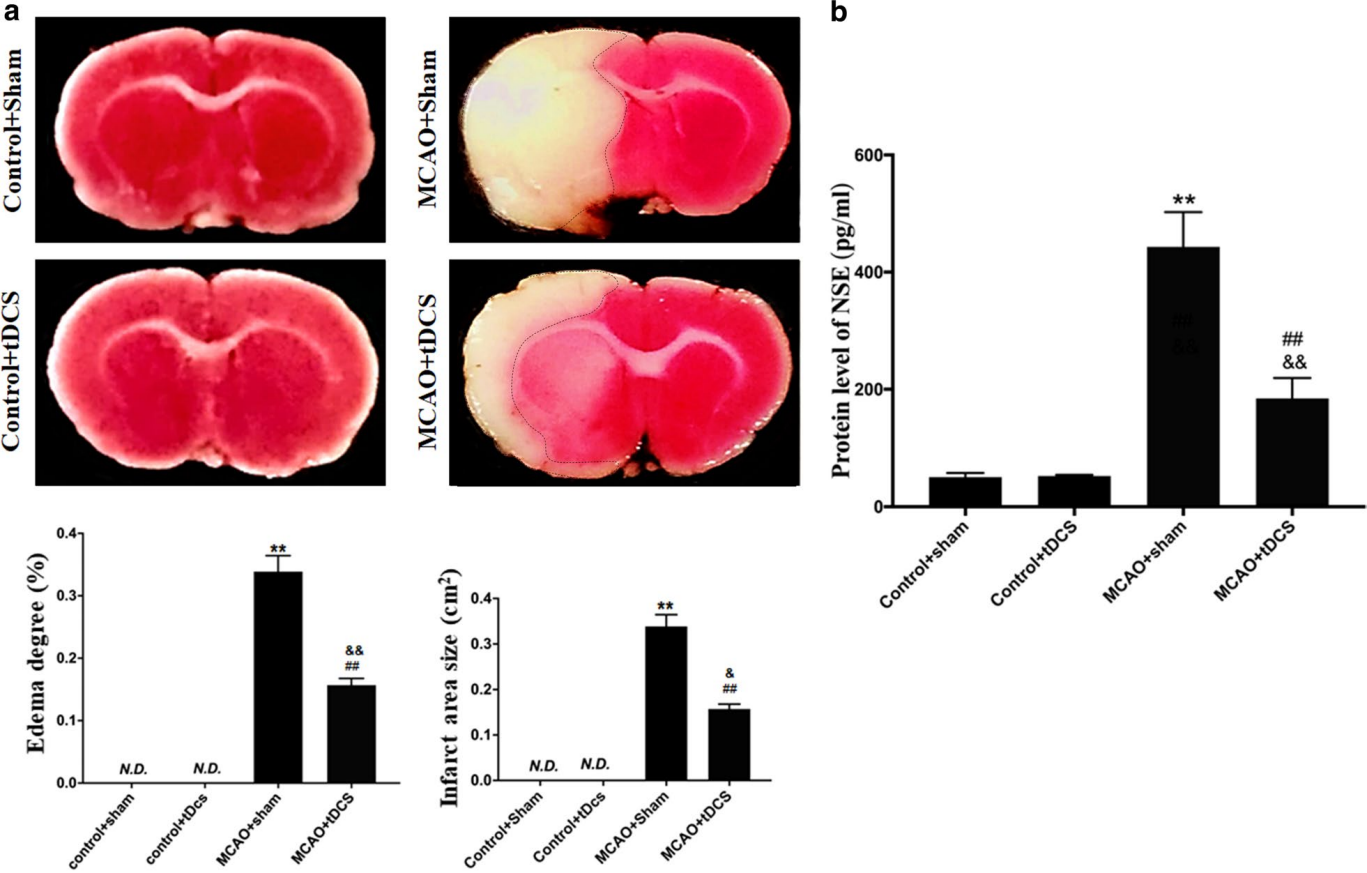
tDCS alleviated the brain damage induced by MCAO operation. **a** TTC staining and semiquantitative analysis. **b** The level of NSE in serum. Data are presented as the mean ± SEM from three independent experiments, and representative images are shown. ^**^P < 0.01, MCAO + Sham *vs* Control + Sham; ^##^*P* < 0.01, MCAO + tDCS *vs* Control + tDCS; ^&&^*P *< 0.01 and ^&^*P* < 0.05, MCAO + Sham *vs* MCAO + tDCS

In this study, the area of brain infarct induced by MCAO operation was visualized by using Triphenyl tetrazolium chloride (TTC) staining. As shown in Fig. 2a and Additional file 3: Table S3, the normal brain tissue was rose-red while the infarct tissue was white. No infarct and brain edema were found in the two control groups. After MCAO operation, the brain infarct was found in part of cortex and striatum ipsilateral to MCAO. Compared with MCAO + Sham group, the area of brain infracts and the degree of brain edema score significantly decreased in MCAO + tDCS group on POD 3 (*t* = 5.646, *P* = 0.023, infract area; *t* = 11.356, *P* = 0.002, edema score).

Neuron-specific Enolase (NSE) is one of the usually used index for brain damage evaluation and prognosis prediction. As shown in Fig. 2b and Additional file 4: Table S4, there was no significant difference in NSE level between Control + Sham group and Control + tDCS group (*t* = 0.043, *P* = 0.967). Compared with two control groups, the level of NSE in serum significantly increased after MCAO operation (*t* = 8.004, *P* = 0.000, MCAO + Sham vs Control + Sham; *t* = 7.962, *P* = 0.000, MCAO + tDCS vs Control + tDCS), suggesting that MCAO induced obvious brain damage in MCAO groups. Compared with MCAO + Sham group, the level of serum NSE in MCAO + tDCS group significantly decreased (*t* = 5.275, *P* = 0.001), which indicated that tDCS alleviated the brain damage induced by MCAO operation.

### tDCS alleviated the brain morphological damage induced by MCAO operation

**Fig. 3:**
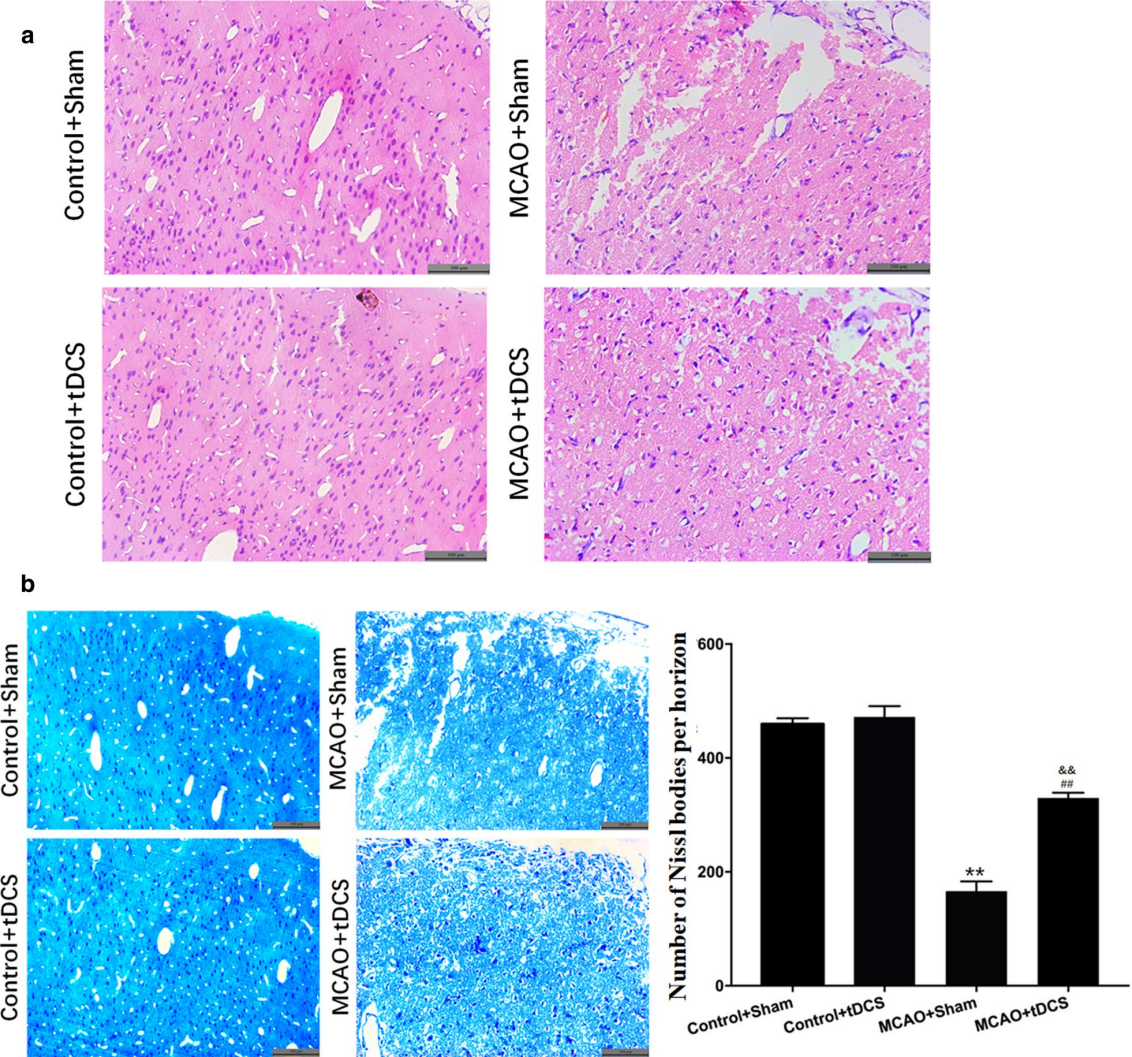
tDCS alleviated the brain morphological damage induced by MCAO operation. **a** The result of hematoxylin and eosin (HE) staining; **b** Nissl staining and semiquantitative analysis. Data are presented as the mean ± SEM from at least three independent experiments, and representative images are shown. ^**^P < 0.01, MCAO + Sham *vs* Control + Sham; ^##^*P* < 0.01, MCAO + tDCS *vs* Control + tDCS; ^&&^*P *< 0.01, MCAO + Sham *vs* MCAO + tDCS. Scale bar = 100 μm

As shown in Fig. 3, the morphology of CIP was obviously altered by MCAO operation on POD 3, which accompanied with disordered arrangement of cells, local degeneration and necrosis. Compared with MCAO + Sham group, the morphology of CIP in MCAO + tDCS group was improved. No differences were found in the morphology of cortex and the number of Nissl bodies between two control groups (*t* = 1.393, *P* = 0.183). Compared with control group, the number of Nissl bodies significantly decreased in CIP in MCAO groups (*t* = 31.882, *P* = 0.000, MCAO + Sham vs Control + Sham; *t* = 15.473, *P* = 0.000, MCAO + tDCS vs Control + tDCS; Fig. 3b and Additional file 5: Table S5). After tDCS treatment, the number of Nissl bodies obviously increased in MCAO + tDCS group, compared with that in MCAO + Sham group (*t* = 17.802, *P* = 0.000). These data suggested that tDCS could alleviate the brain morphological damage induced by MCAO operation.

### tDCS inhibited apoptosis induced by MCAO operation in CIP

**Fig. 4:**
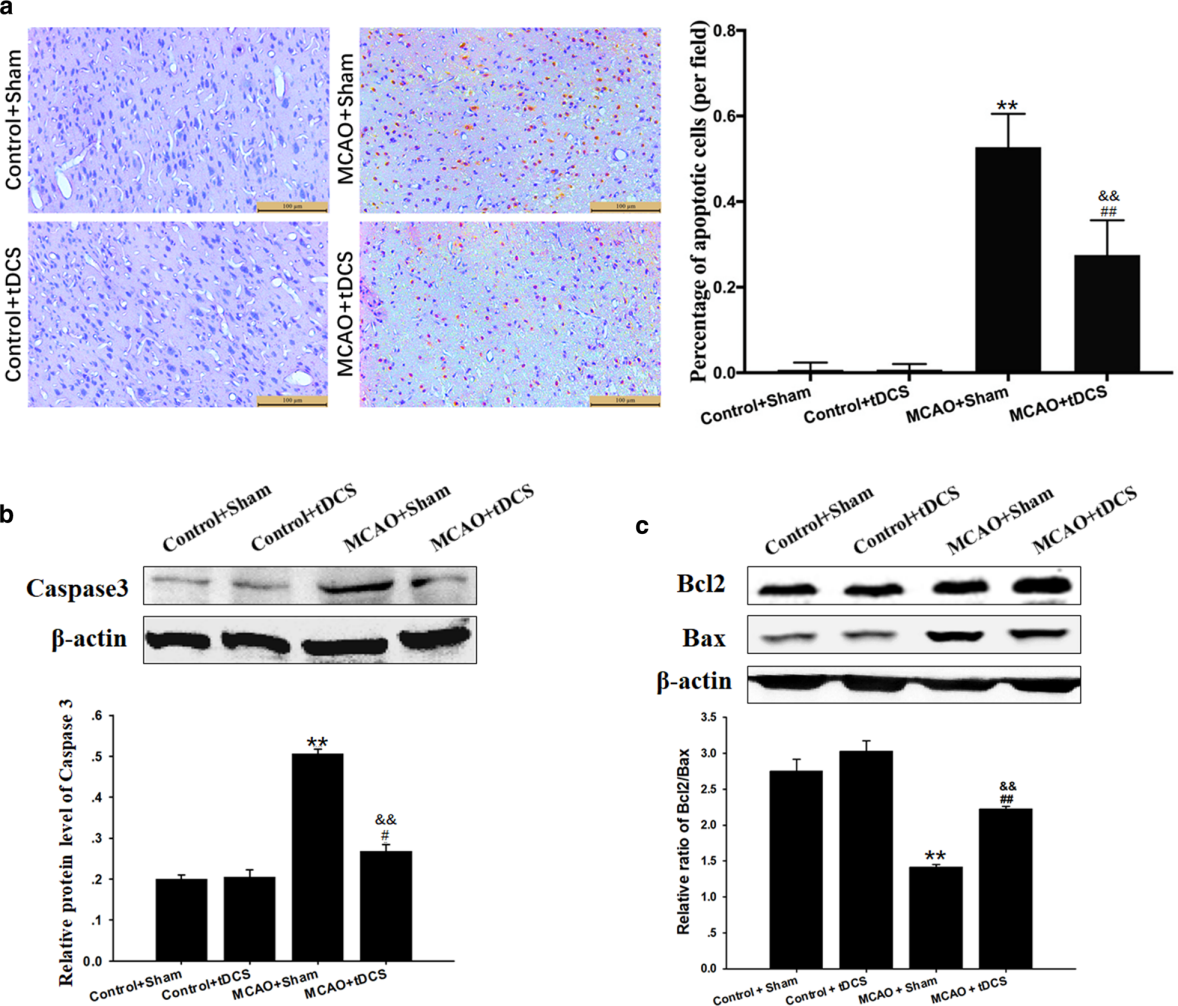
tDCS inhibited apoptosis induced by MCAO operation in CIP. **a** TUNEL staining and semiquantitative analysis; **b** The level and the semiquantitative analysis of Caspase 3; **c** The level of Bcl2 and Bax and the semiquantitative of Bcl2/Bax ratio. Data are presented as the mean ± SEM from at least three independent experiments, and representative images are shown. ^**^P < 0.01, MCAO + Sham *vs* Control + Sham; ^##^*P* < 0.01 and ^#^*P* < 0.05, MCAO + tDCS *vs* Control + tDCS; ^&&^*P *< 0.01, MCAO + Sham *vs* MCAO + tDCS. Scale bar = 100 μm

As shown in Fig. 4a and Additional file 6: Table S6, few terminal dexynucleotidyl transferase-mediated dUTP nick end labeling (TUNEL) positive cells were found in cortex of both two control groups. On POD 3 after MCAO operation, the percentage of TUNEL positive cells significantly increased in CIP, compared with two control groups respectively (*t* = 10.552, *P* = 0.000, MCAO + Sham vs Control + Sham; *t* = 4.281, *P* = 0.001, MCAO + tDCS vs Control + tDCS). And the apoptotic index significantly decreased in MCAO + tDCS group, compared with MCAO + Sham group (*t* = 6.261, *P* = 0.000).

The results of western blot (WB) showed that the protein level of Caspase 3 in CIP was up-regulated after MCAO operation, compared with that of two control groups (*t* = 15.752, *P* = 0.00, MCAO + Sham vs Control + Sham; *t* = 3.265, *P* = 0.046, MCAO + tDCS vs Control + tDCS), which was significantly reduced after tDCS treatment in MCAO + tDCS group, compared with MCAO + Sham group (*t* = 12.224, *P* = 0.000; Fig. 4b, and Additional file 7: Table S7 and Additional file 8: Figure S1). Additionally, the ratio of Bcl2/Bax in CIP significantly decreased after MCAO operation (*t* = 8.347, *P* = 0.000, MCAO + Sham vs Control + Sham; *t* = 5.025, *P* = 0.000, MCAO + tDCS vs Control + tDCS), and tDCS dramatically increased this ratio in MCAO + tDCS group, compared with MCAO + Sham group (*t* = 5.033, *P* = 0.001; Fig. 4c). These data above indicated that tDCS could inhibit apoptosis induced by MCAO operation.

### tDCS inhibited the activation of astrocyte and microglia induced by MCAO operation in CIP

**Fig. 5:**
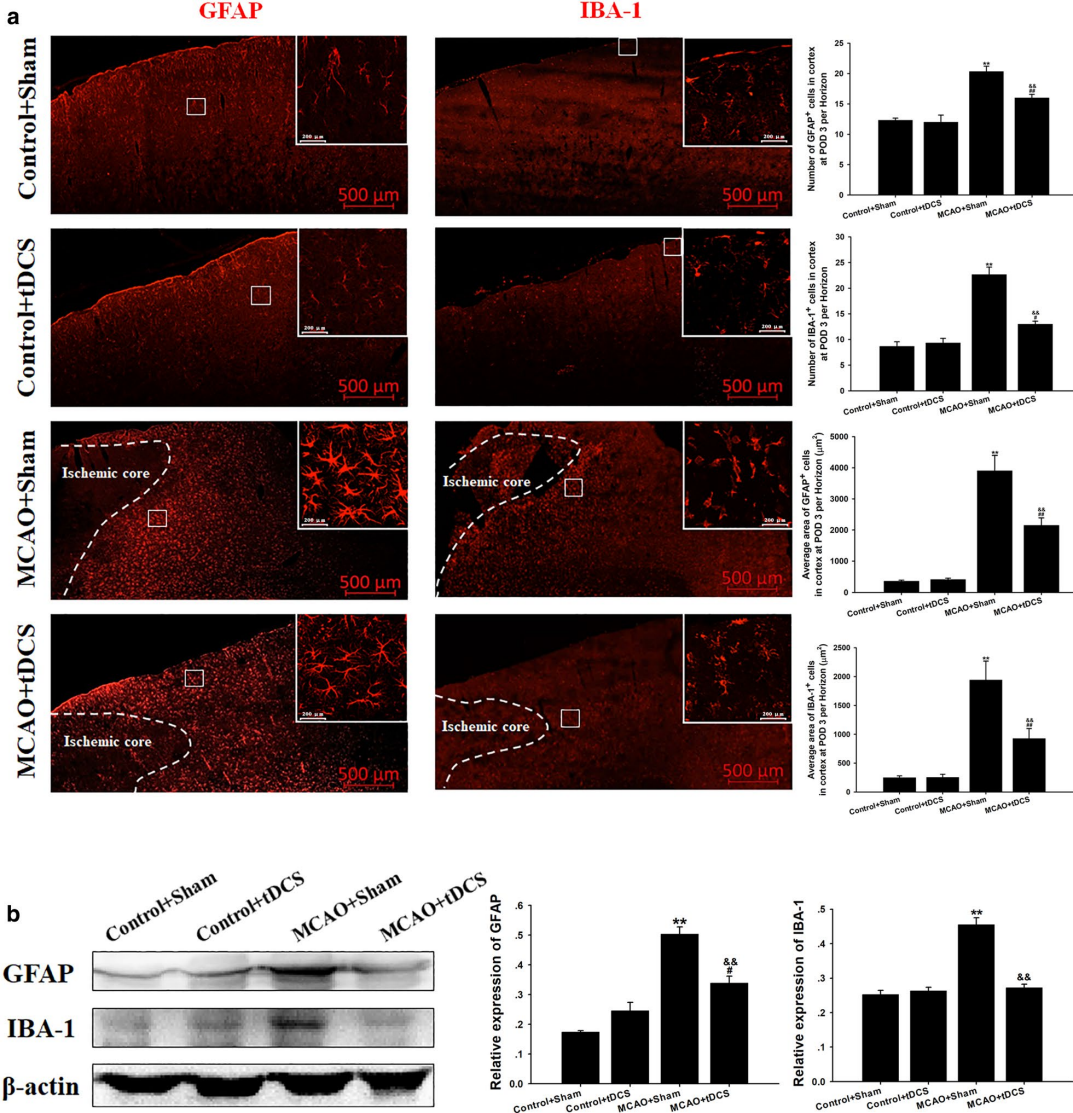
tDCS inhibited the activation of astrocyte and microglia induced by MCAO operation in CIP. **a** Immunohistochemistry (IHC) of GFAP^+^ and IBA-1^+^ cells; **b** The level and the semiquantitative analysis of GFAP and IBA-1. Data are presented as the mean ± SEM from at least three independent experiments, and representative images are shown. ^**^P < 0.01, MCAO + Sham *vs* Control + Sham; ^##^*P* < 0.01 and ^#^*P* < 0.05, MCAO + tDCS *vs* Control + tDCS; ^&&^*P *< 0.01, MCAO + Sham *vs* MCAO + tDCS. Scale bar = 500 μm for macrographs and scale bar = 200 μm for micrographs

As shown in Fig. 5a, and Additional file 9: Table S8 and Additional file 10: Table S9, both the number and body size of GFAP^+^ and of IBA-1^+^ cells significantly increased in CIP after MCAO operation, compared with two control groups (GFAP^+^ body size: *t* = 7.608, *P* = 0.000, MCAO + Sham vs Control + Sham; *t* = 3.577, *P* = 0.001, MCAO + tDCS vs Control + tDCS; IBA-1^+^ body size: *t* = 6.227, *P* = 0.000, MCAO + Sham vs Control + Sham; *t* = 2.327, *P* = 0.024, MCAO + tDCS vs Control + tDCS; GFAP^+^ number: *t* = 7.077, *P* = 0.000, MCAO + Sham vs Control + Sham; *t* = 3.539, *P* = 0.008, MCAO + tDCS vs Control + tDCS; IBA-1^+^ number: *t* = 9.900, *P* = 0.000, MCAO + Sham vs Control + Sham; *t* = 2.593, *P* = 0.032, MCAO + tDCS vs Control + tDCS). Those indexes above significantly decreased in MCAO + tDCS group, compared with that in MCAO + Sham group (GFAP^+^ body size: t = 3.777, P = 0.001; IBA-1^+^ body size: t = 3.841, P = 0.000; GFAP^+^ number: t = 3.833, P = 0.005; IBA-1^+^ number: t = 6.835, P = 0.000). Additionally, the expression of GFAP and IBA-1 was up-regulated in CIP of MCAO group, compared with that in two control groups (GFAP^+^: *t* = 10.623, *P* = 0.000, MCAO + Sham vs Control + Sham; *t* = 3.007, *P* = 0.017, MCAO + tDCS vs Control + tDCS; IBA-1^+^: *t* = 10.045, *P* = 0.000, MCAO + Sham vs Control + Sham; *t* = 0.461, *P* = 0.656, MCAO + tDCS vs Control + tDCS; Fig. 5b, and Additional file 8: Figure S1 and Additional file 11: Table S10). The over-expressions of GFAP and IBA-1 induced by MCAO was also alleviated by tDCS (GFAP^+^: t = 5.301, P = 0.001; IBA-1^+^: t = 9.067, P = 0.000). These results suggested that tDCS could inhibit the activation of astrocyte and microglia induced by MCAO operation. No differences were found in morphology and protein expression of GFAP and IBA-1 between Control + Sham group and Control + tDCS group (GFAP^+^ body size: *t* = 0.110, *P* = 0.914; GFAP^+^ number: *t* = 0.295, *P* = 0.776; GFAP^+^ expression: *t* = 2.310, *P* = 0.175; IBA-1^+^ body size: *t* = 0.012, *P* = 0.990; IBA-1^+^ number: *t* = 0.471, *P* = 0.650; IBA-1^+^ expression: *t* = 0.521, *P* = 0.952).

### tDCS decreased the level of inflammatory factors induced by MCAO operation in CIP

**Fig. 6:**
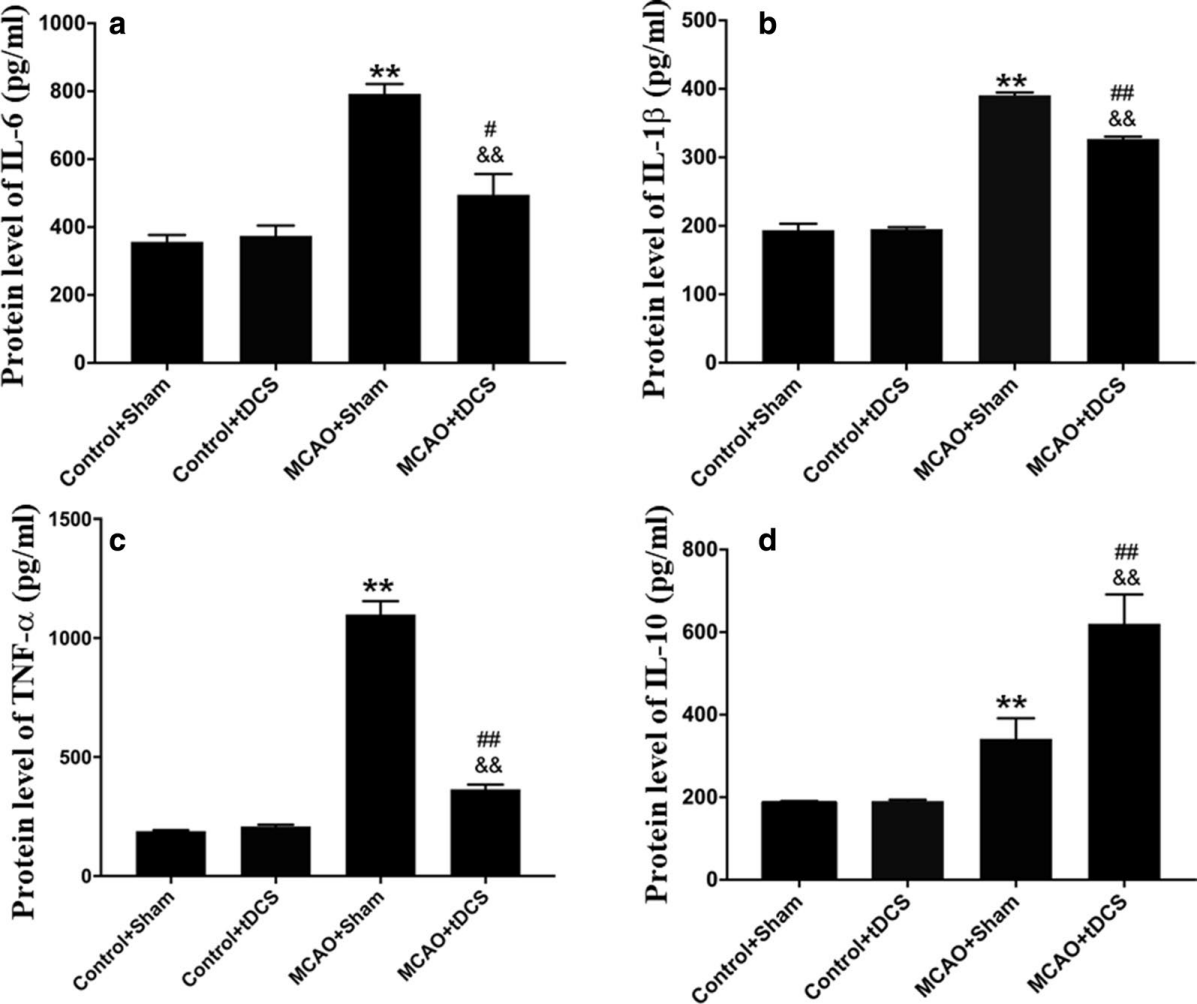
tDCS decreased the level of inflammatory factors induced by MCAO operation in CIP. The quantification of IL-6 (**a**), IL-1β (**b**), TNF-α (**c**) and IL-10 (**d**) in CIP. Data are presented as the mean ± SEM from three independent experiments, and representative images are shown. ^**^P < 0.01, MCAO + Sham *vs* Control + Sham; ^##^*P* < 0.01 and ^#^*P* < 0.05, MCAO + tDCS *vs* Control + tDCS; ^&&^*P *< 0.01, MCAO + Sham *vs* MCAO + tDCS

Compared with two control groups, the level of pro-inflammatory factors (i.e. IL-6, IL-1β and TNF-α) and anti-inflammatory factors (i.e. IL-10) significantly increased in CIP on POD 3 (IL-6: *t* = 8.050, *P* = 0.000, MCAO + Sham vs Control + Sham; *t* = 2.227, *P* = 0.057, MCAO + tDCS vs Control + tDCS; IL-1β: *t* = 18.732, *P* = 0.000, MCAO + Sham vs Control + Sham; *t* = 12.517, *P* = 0.000, MCAO + tDCS vs Control + tDCS; TNF-α: *t* = 50.167, *P* = 0.000, MCAO + Sham vs Control + Sham; *t* = 6.026, *P* = 0.000, MCAO + tDCS vs Control + tDCS; IL-10: *t* = 4.350, *P* = 0.011, MCAO + Sham vs Control + Sham; *t* = 12.109, *P* = 0.000, MCAO + tDCS vs Control + tDCS). After tDCS treatment, the level of pro-inflammatory factors significantly decreased and anti-inflammatory factor significantly increased in CIP of MCAO + tDCS group, compared with that in MCAO + Sham group (IL-6: *t* = 5.494, *P* = 0.003; IL-1β: *t* = 6.0715, *P* = 0.000; TNF-α: *t* = 43.12, *P* = 0.000; IL-10: *t* = 7.874, *P* = 0.000), which indicated that tDCS could inhibit neuroinflammatory induced by MCAO operation. No differences were found in both two control groups (IL-6: *t* = 0.329, *P* = 0.987; IL-1β: *t* = 0.144, *P* = 0.889; TNF-α: *t* = 1.020, *P* = 0.337; IL-10: *t* = 0.114, *P* = 0.999; Fig. 6 and Additional file 12: Table S11).

## Discussion

tDCS has been reported to be beneficial for the alleviation of neuropsychiatric and neurological conditions, and the balance modulation of some excitatory and inhibitory neurotransmitters, such as N-methyl-d-aspartic acid (NMDA) and γ-aminobutyric acid (GABA) was supposed to be involved in it [12]. In addition, it was found that tDCS could induce an elevation in astrocytic Ca^2+^, which has subsequently been demonstrated to be important for cortical plasticity [13]. Similar findings in tDCS induced Na^+^ modulation have also been reported [12]. As a result, the membrane potential of neurons could be depolarized or hyperpolarized, and further regulate the disease progression through the manner of neuronal excitability toxicity [14]. Besides, tDCS was proved to regulate neural networks [15]. Through tDCS-induced synchrony and resonance of subcortical networks, the functional connectivity among different brain regions would be enhanced, which may greatly assist stroke rehabilitation [16].

In the present study, we adopted the stimulation parameters (500 μA, 15 min, cathodal) as reported in a previous study [17], and the efficacy of tDCS on neuroprotection has been investigated. It was found that the overall trend of neurological deficit recovery was significantly promoted by tDCS treatment, although the difference of mNSS was rather small and even not significant at POD 14 between MCAO + Sham and MACO + tDCS. On one hand, it might attribute to the difference of statistic methods, since the data of single timepoint were usually analyzed by one-way ANOVA and post hoc LSD-t, while the overall outcomes were usually analyzed by repeated-measures ANOVA. On the other hand, the good self-healing capability of rats might also be involved in it. Besides, the area of cerebral infarct and the degree of cerebral edema induced by MCAO significantly decreased after tDCS on POD 3. Additionally, tDCS treatment also significantly decreased the level of NSE, a vital index for brain injury evaluation [18], in the serum of MCAO rat on POD 3. The above results indicated that tDCS treatment may play a neuroprotective role at the early stage of ischemic stroke, which was consistent with Li’s report [19], they found that at the acute phase of ischemic stroke, cathode tDCS (250 μA, 20 min on–20 min off –20 min on) could significantly decrease the area of cerebral infarct and exert a neuroprotective effect.

As we known, ischemia and hypoxia could directly cause irreversibly damage to cells in the core of the infarct, which cannot be rescued over 6 h after onset [20]. However, there are still a large number of reversibly damaged cells in the CIP [21], which is the focus of modern therapy for ischemic stroke. The number of Nissl bodies would be reduced, or even drained when neurons were under the pathological circumstance. In the present study, we found that tDCS not only improved the morphology of brain tissue, but also reduced the percentage of apoptotic cells in CIP after ischemic stroke. Considering the results of Nissl staining and TUNEL staining, we speculate that tDCS may protect neurons of CIP from apoptosis in ischemic conditions. It has been well documented that the ratio of Bcl2/Bax [22, 23], as well as Caspase 3, play important roles in apoptosis [24]. In this study, the changes of Caspase 3 and Bcl2/Bax were consistent with the results of TUNEL staining, indicating that the anti-apoptosis effect of tDCS was involved in the neuroprotection.

Astrocyte and microglia play a vital role in the pathophysiological process of neuroinflammatory response [25], which is one of the leading causes of secondary injury after stroke. Once astrocyte and microglia were activated, proliferation and morphological changes would take place, with the characteristic of enlarged cell body, thickened protuberance and deepened staining [26]. Additionally, activated glial cells can release a variety of toxic substances, such as inflammatory cytokines, nitric oxide and reactive oxygen species, and then resulted in neural apoptosis and aggravate ischemic damage [27]. It was reported that tDCS could reduce infarct area and apoptosis by affecting microglia polarization and inhibiting inflammatory response at the hyper-acute phase of stroke [28]. In the present study, we found that tDCS decreased the number and body size of IBA-1^+^ cells in CIP of MCAO rats on POD 3, as well as the protein level of IBA-1, which indicated that tDCS could suppress the activation of microglia at the acute phase of ischemic stroke [29]. However, Braun et al. [17] reported that tDCS administration increased the number of M1 microglia after ischemic injury on POD 13, while M2 microglia was not affected. Notably, pro-inflammatory action is regulated by M1 microglia and M2 microglia participates in the anti-one [30]. Therefore, tDCS may exert diverse effects on microglia at different phases of ischemic stroke. Astrocyte is another kind of vital cells that could modulate neuroinflammatory response [31], and affect the neural plasticity (the basis for functional recovery) [32]. It was reported that if astrocyte activation could not be controlled at a proper level in time, reactive gliosis can exert inhibitory effects on several aspects of neuroplasticity and CNS regeneration [33]. Thus, modulating astrocyte function might become an attractive opportunity for novel therapeutic interventions. In this study, we found that tDCS significantly inhibited the activation of astrocyte in CIP under the pathological condition of ischemic stroke. Monai et al. reported that tDCS could regulate astrocyte and cortical plasticity [13]. Taken together, we speculate that tDCS may promote neural plasticity to accelerate functional recovery via the modulation of astrocyte activation.

Inflammatory cytokines released from activated glial cells can deteriorate the microenvironment and aggravate apoptosis in CIP [34, 35]. It was reported that tDCS could relieve the hyperalgesia induced by complete Freund’s adjuvant [36] and chronic constriction injury [37] through regulating the level of IL-1β, IL-10, and TNF-α in the cerebral cortex. In this study, we found that tDCS administration significantly reduced the level of pro-inflammatory cytokines (i.e. IL-1β, IL-6 and TNF-α) and increased the level of anti-inflammatory factor (i.e. IL-10) in the CIP, which was consistent with previous study [38]. Taking the results of morphology and cytokines at POD 3 together, we speculated that tDCS could inhibit the neuroinflammation response in CIP and result in a neuroprotective effect in the early stroke stage, which would accelerate rehabilitation of behavioral function in mNSS at POD 6 between MCAO + Sham and MCAO + tDCS. Notably, there was a disproportionate change between behavioral performance and other measurements after tDCS treatment. Specifically, the reduction of MCAO-induced neurological deficit was much smaller than those of other indexes, such as glial activation, neuroinflammation, and apoptosis. As we know, mNSS is consisted of three parts, including motor test, sensory test and abnormal reflection, which are regulated by different brain regions. In this study, we mainly focused on the cerebral ischemic penumbra which was corresponding to the motor cortex M1 and M2. Therefore, a non-point-to-point manner may lead to a disproportionate change.

Though we have preliminarily demonstrated the efficacy of tDCS in the early stage of ischemic stroke, there is a limitation need to be addressed. In this study, some clinical indexes, such as temperature measurement and other physiological parameters were not detected, which is also vital for future clinical application of tDCS. Therefore, although we have got some evidences of neuroinflammatory response and neuroprotection effect, much future work are necessary to explore the mechanism of tDCS’s therapeutic effect on stroke.

## Conclusion

Our results suggested that under this experimental condition (500 μA, 15 min, once per day), tDCS treatment could significantly accelerate the recovery of neurologic deficit and exert neuroprotective effect after ischemic stroke. The inhibition of neuroinflammatory and apoptosis in CIP may be involved in the process.

## Materials and methods

### Animals

Male Sprague–Dawley rats (230–250 g), obtained from the Center of Experimental Animal in the Fourth Military Medical University (Xi’an, China), were housed in plastic cages (4 animals per cage) with free access to food and water in a temperature-controlled room at 22–25 °C on a 12-h light/dark cycle. All of the experiments reported in this study were conducted according to an experimental protocol of the Animal Use and Care Committee for Research and Education of the Fourth Military Medical University. All efforts were made to minimize the animals’ suffering and the number of animals used. For euthanasia, the rats were deeply anesthetized with 1% pentobarbital 8 sodium (60 mg/kg i.p.).

### Surgery

**Fig. 7:**
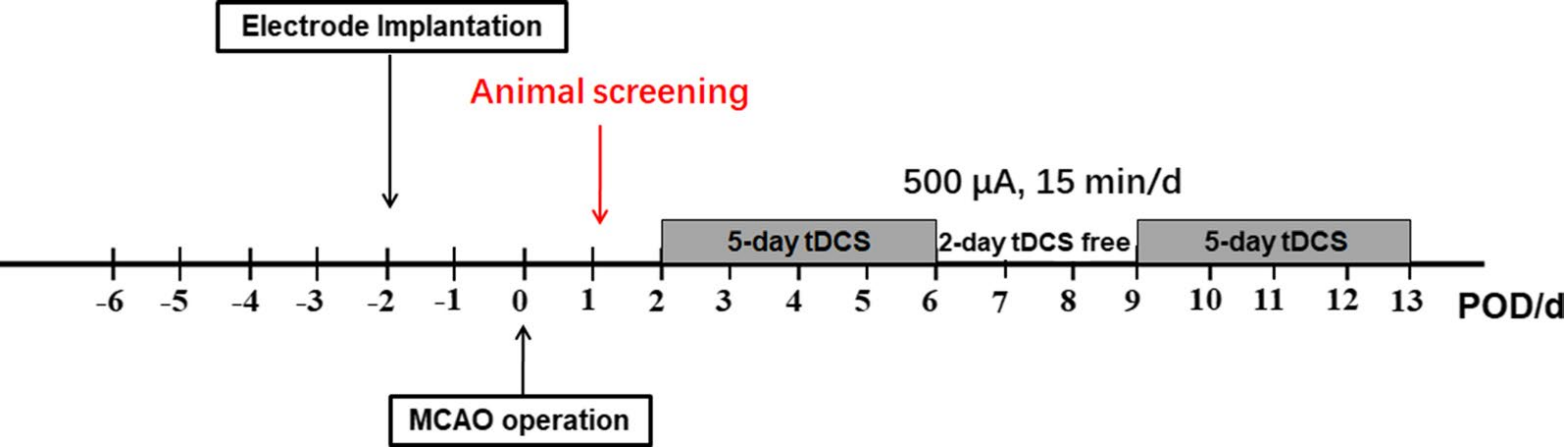
Schematic presentation of the treatment regime of the animals

Two days before the MCAO operation, all rats were accepted epicranial electrode implantation (Fig. 7). The rats were anesthetized with 1% pentobarbital sodium (45 mg/kg, intraperitoneally [i.p.]) and fixed on the brain stereotaxic apparatus (RWD Life Science, China). After removal of the scalp and underlying tissues, a homemade epicranial electrode with a defined contact area of 3.5 mm^2^ was mounted on the intact skull at the coordinates bregma AP + 2.0 mm and ML + 2.0 mm with glass ionomer dental cement (Ketac Cem, ESPE Dental AG, Seefeld, Germany). The electrode was remained in place during the whole experiment.

After epicranial electrode implantation, half of the rats with implanted electrodes were undergone a transient focal ischemia operation by temporary MCAO as described previously [39]. In addition, the other half of animals with implanted electrodes underwent a sham MCAO operation (i.e., all steps of the operation were performed except the MCAO).

### Groups and tDCS treatment

24 h after MCAO operation, the severity degree of neurological deficits was evaluated by a five-point deficit score as previously reported [39]. After ruling out dead or severely injured rats, 55 animals with moderate neurological deficits (grade 2 of 4) were selected from 120 animals that underwent the MCAO operation and were randomly divided into two experimental groups (Additional file 13: Table S12): MCAO + tDCS group (n = 28) and MCAO + Sham group (n = 27). The other 54 animals that underwent a sham MCAO operation were randomly divided into two control groups: Control + tDCS (n = 27) and Control + Sham (n = 27).

Prior to the tDCS, a homemade epicranial electrode fixed on the head of rats was soaked with saline solution to reduce the impedance and a rubber plate electrode (contact area of 7.0 cm^2^) was fixed on the chest by a corset. Then, the circuit was connected and tDCS was generated continuously with a constant current stimulator (PMP18-3TR, Kikusui, Japan). During the tDCS treatment, the average charge density was 128,571 C/m^2^. The animals in two tDCS groups (MCAO + tDCS and Control + tDCS) underwent 5 consecutive days of cathodal tDCS (500 μA, 15 min, once per day) treatment beginning on POD 2 followed by a 2-day tDCS-free interval and then 5 days of tDCS again, totally 10 days, which parameter has been frequently adopted in tDCS therapeutic studies [17, 40, 41]. The animals in two sham tDCS groups (MCAO + Sham and Control + Sham) were only connected to the tDCS stimulator for 15 min but without continuous output current. The stimulating current was ramped up or down to the expected intensity within 10 s, so as to avoid discomfort to experimental animals caused by sudden rise and sudden drop of current intensity. Animals were awake during electrical stimulation.

### Bodyweight monitoring

To investigate the effect of tDCS on the general health of the animals after MCAO operation, the body weight was detected every 2 days and the first detection was performed 2 days before MCAO operation (n = 12 for each group). The detection time was fixed at 3:00 PM.

### mNSS test

mNSS is a commonly used index to evaluate the severity degree of neurological deficit [42], which consists of motor test, sensory test, and abnormal reflection. In this study, mNSS was performed immediately after bodyweight detection and it was carried out by the researchers who were blind to the experimental design. The animals tested with mNSS were the same in the bodyweight monitor.

### TTC staining

TTC is a redox indicator to distinguish infarct tissues from normal tissue. In the present study, immediately after tDCS treatment on POD 3, the brains were rapidly removed under deep anesthesia (1% pentobarbital sodium, 60 mg/kg i.p.) and frozen at −20 °C for 15 min (n = 6 for each group). Then, the brain was sliced coronally at 2-mm thick sections using a special brain matrix for rats (RWD Life Science, China). After that, the sections were stained with 2% TTC (Sigma-Aldrich) at 37 °C for 30 min. Finally, the brain slices were photographed by a digital camera and analyzed using Image J software. The area of infarct tissue was calculated by measuring the unstained area of each slice, and the brain edema score was calculated as reported previously [43].

### Hematoxylin and eosin (HE) staining and Nissl staining

HE and Nissl staining have been performed as previously reported [41]. Briefly, immediately after tDCS treatment on POD 3, the rats were anesthetized (1% pentobarbital sodium, 60 mg/kg i.p.) and transcardially perfused with 200 ml of 5 mM sodium phosphate-buffered 0.9% (w/v) saline (PBS, pH 7.2–7.4) followed by 500 ml of 4% paraformaldehyde in phosphate buffer (n = 6 for each group), then the brains were rapidly removed. After being dehydrated in ethanol and defatted in xylene, the brain was embedded in paraffin and 4 μm thick horizontal slices were obtained by using a rotary microtome (Leica RM2135, Leica Biosystems, Germany). For HE staining, the sections were dipped in hematoxylin for 3 min, washed in running tap water and destained in hydrochloric acid alcohol for several seconds. The sections were washed again and then dipped in eosin for 15 s. They were subsequently dehydrated in an alcohol gradient, cleared in xylene, and coverslipped. For Nissl staining, the sections were dipped in 1% toluidine blue for 30 min at 45 °C, and differentiated in 75% alcohol for seconds, then rinsed quickly in distilled water. Digital images were acquired using an inverted microscope (DMI4000B, Leica, Leica Biosystems, Germany).

### TUNEL staining

The paraffin blocks of the brain used for HE and Nissl staining were also used for apoptotic cell evaluation. The TUNEL kit (Rothe, Basel, Swetizerland) was used to detect the apoptotic cells and the experiment was performed according to the manufacturer’s instructions. The digital photographs were acquired with Leica DMI4000B. The percentage of apoptotic cells was calculated by the researchers who were blind to the experimental design.

### Immunohistochemical staining (IHC)

The morphology of microglia and astrocyte has been detected as previously reported [44]. The brains were harvested as described in HE and Nissl staining part, and immersed in 30% (w/v) sucrose at 4 °C until they sank to the bottom (n = 6 for each group). Serial coronal, 25 μm brain sections were prepared using a cryotome (Leica CM1800; Heidelberg, Germany) and then stored at –20 °C until use. During IHC, the following primary antibodies were used: rabbit anti-IBA-1 (1:500, cat# 019-19741, Wako, Osaka, Japan), a biomarker of microglia; polyclonal rabbit anti-GFAP (1:1000, cat# 16825-1-AP, Proteintech, Wu Han, China), a biomarker of astrocyte. The sections were incubated overnight at 4 °C and rewarmed at room temperature for 40 min. After being washed with 0.01 M PBS, the secondary antibody was added to the sections: Alexa Fluor 594-conjugated donkey anti-rabbit IgG (1:500, cat# 127803, Jackson Immuno Research Labs, West Grove, PA). After incubation with secondary antibodies for 3 h at room temperature, the sections were mounted onto glass slides. Digital images were obtained using a confocal laser-scanning microscope (LSM 800, Zeiss, Oberkochen, Germany).

### Elisa

The rats were sacrificed immediately after tDCS treatment on POD 3 as described in the TTC staining part. Blood samples (5 ml) were collected from the rat heart. Then, the CIP tissue of the brain was immediately separated and frozen. The serum was collected from blood after centrifugation at 1500 rpm for 15 min at 4 °C. The CIP samples were weighed and homogenized in radioimmunoprecipitation assay lysis buffer supplemented with the proteinase inhibitor phenylmethylsulfonyl fluoride using a homogenizer device (Leica, Heidelberg, Germany). The supernatant was acquired after centrifugation at 12,000 rpm for 15 min at 4 °C. The serum was used for NSE enzyme-linked immunosorbent assay (ELISA) detection (Elabscience, China), and supernatant of CIP was used for inflammatory cytokines ELISA detection (i.e. IL-6, IL-1β, IL-10, TNF-α; ImmunoWay, USA). Experiments were performed according to the manufacturer’s instructions and the absorbance was measured by using an enzyme standard instrument at 450 nm (Bio-Rad, USA).

### Western blot assay (WB)

WB is a commonly used method to evaluate the change of protein [45]. In present study, protein expression levels in CIP were detected by WB on POD 3 (n = 6 for each group). The supernatant of CIP tissue was collected as described in the ELISA part. The total protein levels were quantified by a bicinchoninic acid assay kit (Beyotime Biotechnology, Jiangsu, China). The following antibodies were used: rabbit anti-IBA-1 (1:500, cat# 019-19741, Wako, Osaka, Japan); polyclonal rabbit anti-GFAP (1:1000, cat# 16825-1-AP, Proteintech, Wu Han, China); polyclonal rabbit anti-Caspase 3 (1:3000, cat# 19677-1-AP, Proteintech, Wu Han, China); polyclonal rabbit anti-Bax (1:5000, cat# 50599-2-Ig, Proteintech, Wu Han, China); polyclonal rabbit anti-Bcl2 (1:1000, cat# ab59348, Abcam, United Kingdom); monoclonal mouse anti-β-actin (1:3000, cat# 7G6, CMCTAG, USA), and corresponding secondary antibodies. The blots were visualized using an enhanced chemiluminescence reagent (Millipore, MA, USA) and captured using ChemiDoc^TM^ MP System (Bio-Rad, UK). For quantification, the target protein levels were normalized to the β-actin levels and were expressed as relative fold changes. The densities of protein blots were analyzed using Image J Software by the researchers who were blind to the experimental design.

### Statistical analysis

Repeated-measures analysis of variance (ANOVA) was used for the data from the bodyweight monitoring and mNSS. Data from IHC, WB and ELISA were analyzed with one-way ANOVA followed by the least-significant difference tests (LSD-t) for post hoc analysis. All data are presented as mean ± SEM, and all statistical analyses were performed using SPSS software version 20.0 (SPSS Inc., Chicago, IL, USA). *P* < 0.05 was considered as statistically significant.

## Supplementary information

**Additional file 1: Table S1.** The results of body weight (g).

**Additional file 2: Table S2.** The results of mNSS score.

**Additional file 3: Table S3.** The results of TTC staining

**Additional file 4: Table S4.** Protein level of NSE (pg/ml).

**Additional file 5: Table S5.** The number of Nissl bodies.

**Additional file 6: Table S6.** The percentage of apoptotic cells.

**Additional file 7: Table S7.** The level of apoptosis related protein.

**Additional file 8: Figure S1.** Related western blot data in triplicate.

**Additional file 9: Table S8.** The number of GFAP^+^ and IBA-1^+^ cells.

**Additional file 10: Table S9.** The size of GFAP^+^ and IBA-1^+^ cells.

**Additional file 11: Table S10.** The protein level of GFAP and Iba1.

**Additional file 12: Table S11.** The level of inflammatory factors.

**Additional file 13: Table S12.** Animal distribution.

## Data Availability

Raw data has been provided as additional files.

## Abbreviations

tDCS: Transcranial direct current stimulation
MCAO: Middle cerebral artery occlusion
CIP: Cerebral ischemic penumbra
POD: The day post operation
NSCs: Neural stem cells
SEM: Standard error of mean
mNSS: Modified neurological deficit score
TTC: Triphenyl tetrazolium chloride
NSE: Neuron-specific enolase
HE: Hematoxylin and eosin
TUNEL: Terminal dexynucleotidyl transferase-mediated dUTP nick end labeling
WB: Western blot
IHC: Immunohistochemistry
PBS: Phosphate-buffered saline
ELISA: Enzyme linked immunosorbent assay
ANOVA: Analysis of variance
