# Supplementary material for: Cathodal tDCS exerts neuroprotective effect in rat brain after acute ischemic stroke
Source: BMC Neurosci. 2020 May 12;21:21. doi: 10.1186/s12868-020-00570-8 (PMC7216334; doi:10.1186/s12868-020-00570-8)
Supplement: Supplementary file 1 — Additional file 1: Table S1. The results of body weight (g). [file 12868_2020_570_MOESM1_ESM.docx]

**Additional file 1.** The results of body weight (g).

| **Groups** | **Detecting timepoints** | | | | | | |
| --- | --- | --- | --- | --- | --- | --- | --- |
|  | **POD 0** | **POD 2** | **POD 4** | **POD 6** | **POD 8** | **POD 10** | **POD 14** |
| **Control + Sham  (n = 6)** | 274.8 | 281.4 | 293.5 | 300.1 | 311.2 | 325.4 | 330.7 |
|  | 277.3 | 285.6 | 295.1 | 302.1 | 313.2 | 321.6 | 338.3 |
|  | 280.8 | 286.4 | 293.5 | 305.4 | 316.6 | 329.7 | 341.2 |
|  | 279.4 | 284.6 | 291.4 | 295.4 | 307.8 | 312.5 | 321.8 |
|  | 270.7 | 277.9 | 282.7 | 290.1 | 299.6 | 311.4 | 322.5 |
|  | 279.1 | 283.5 | 294.6 | 299.3 | 306.5 | 320.2 | 325.4 |
| **Control + tDCS  (n = 6)** | 275.4 | 281.1 | 290.6 | 295.7 | 318.8 | 323.1 | 337.7 |
|  | 276.4 | 283.3 | 292.5 | 297.6 | 311.7 | 320.5 | 329.8 |
|  | 279.3 | 285.6 | 291.9 | 299.3 | 317.1 | 325.7 | 330.1 |
|  | 272.4 | 277.4 | 285.3 | 300.6 | 313.5 | 319.9 | 331.5 |
|  | 278.8 | 283.5 | 291.6 | 303.7 | 311.3 | 320.4 | 331.6 |
|  | 280.0 | 287.4 | 295.1 | 306.4 | 312.2 | 323.5 | 329.8 |
| **MCAO + Sham  (n = 6)** | 303.0 | 256.8 | 233.0 | 240.7 | 262.3 | 285.6 | 298.2 |
|  | 261.4 | 195.6 | 166.8 | 178.6 | 185.2 | 204.1 | 246.3 |
|  | 266.6 | 224.6 | 243.7 | 243.6 | 276.5 | 302.2 | 312.9 |
|  | 273.8 | 227.3 | 243.0 | 261.5 | 262.1 | 278.6 | 281.5 |
|  | 259.1 | 188.6 | 187.5 | 214.8 | 214.5 | 240.2 | 272.6 |
|  | 283.2 | 240.0 | 275.0 | 286.4 | 281.5 | 307.0 | 298.4 |
| **MCAO + tDCS  (n = 7)** | 284.4 | 233.0 | 285.0 | 302.2 | 310.7 | 334.6 | 344.9 |
|  | 263.0 | 199.7 | 189.9 | 238.6 | 252.1 | 269.8 | 273.8 |
|  | 285.3 | 248.7 | 272.9 | 278.4 | 316.2 | 323.6 | 324.0 |
|  | 277.5 | 239.9 | 255.8 | 282.7 | 282.8 | 316.4 | 319.5 |
|  | 252.7 | 210.4 | 252.5 | 303.8 | 298.3 | 310.7 | 320.0 |
|  | 278.2 | 248.3 | 272.9 | 278.4 | 316.2 | 323.6 | 324.0 |
|  | 302.5 | 287.9 | 303.4 | 309.8 | 318.5 | 325.3 | 356.5 |
