# Supplementary material for: Cathodal tDCS exerts neuroprotective effect in rat brain after acute ischemic stroke
Source: BMC Neurosci. 2020 May 12;21:21. doi: 10.1186/s12868-020-00570-8 (PMC7216334; doi:10.1186/s12868-020-00570-8)
Supplement: Supplementary file 2 — Additional file 2: Table S2. The results of mNSS score. [file 12868_2020_570_MOESM2_ESM.docx]

**Additional file 2.** The results of mNSS score.

| **Groups** | **Detecting timepoints** | | | | | | |
| --- | --- | --- | --- | --- | --- | --- | --- |
|  | **POD 0** | **POD 2** | **POD 4** | **POD 6** | **POD 8** | **POD 10** | **POD 14** |
| **Control + Sham  (n = 6)** | 0 | 0 | 0 | 0 | 0 | 0 | 0 |
|  | 0 | 0 | 0 | 0 | 0 | 0 | 0 |
|  | 0 | 0 | 0 | 0 | 0 | 0 | 0 |
|  | 0 | 0 | 0 | 0 | 0 | 0 | 0 |
|  | 0 | 0 | 0 | 0 | 0 | 0 | 0 |
|  | 0 | 0 | 0 | 0 | 0 | 0 | 0 |
| **Control + tDCS  (n = 6)** | 0 | 0 | 0 | 0 | 0 | 0 | 0 |
|  | 0 | 0 | 0 | 0 | 0 | 0 | 0 |
|  | 0 | 0 | 0 | 0 | 0 | 0 | 0 |
|  | 0 | 0 | 0 | 0 | 0 | 0 | 0 |
|  | 0 | 0 | 0 | 0 | 0 | 0 | 0 |
|  | 0 | 0 | 0 | 0 | 0 | 0 | 0 |
| **MCAO + Sham  (n = 6)** | 0 | 6 | 6 | 5 | 5 | 4 | 4 |
|  | 0 | 7 | 8 | 7 | 5 | 3 | 3 |
|  | 0 | 7 | 6 | 4 | 4 | 4 | 3 |
|  | 0 | 10 | 6 | 5 | 5 | 4 | 3 |
|  | 0 | 9 | 6 | 4 | 4 | 4 | 3 |
|  | 0 | 10 | 6 | 5 | 5 | 5 | 6 |
| **MCAO + tDCS  (n = 7)** | 0 | 10 | 6 | 4 | 4 | 4 | 4 |
|  | 0 | 10 | 9 | 4 | 4 | 3 | 3 |
|  | 0 | 10 | 6 | 2 | 2 | 2 | 2 |
|  | 0 | 8 | 4 | 2 | 2 | 2 | 2 |
|  | 0 | 5 | 5 | 4 | 3 | 3 | 3 |
|  | 0 | 5 | 5 | 3 | 2 | 2 | 1 |
|  | 0 | 6 | 6 | 4 | 4 | 3 | 3 |
