# Supplementary material for: Cathodal tDCS exerts neuroprotective effect in rat brain after acute ischemic stroke
Source: BMC Neurosci. 2020 May 12;21:21. doi: 10.1186/s12868-020-00570-8 (PMC7216334; doi:10.1186/s12868-020-00570-8)
Supplement: Supplementary file 3 — Additional file 3: Table S3. The results of TTC staining [file 12868_2020_570_MOESM3_ESM.docx]

**Additional file 3.** The results of TTC staining.

| **Groups** | **Edema degree** | **Infarct area size** |
| --- | --- | --- |
| **Control + Sham  (n = 3)** | N.D. | N.D. |
|  | N.D. | N.D. |
|  | N.D. | N.D. |
| **Control + tDCS  (n = 3)** | N.D. | N.D. |
|  | N.D. | N.D. |
|  | N.D. | N.D. |
| **MCAO + Sham  (n = 3)** | 0.3445 | 0.2206 |
|  | 0.3612 | 0.1710 |
|  | 0.3110 | 0.1875 |
| **MCAO + tDCS  (n = 3)** | 0.1587 | 0.1116 |
|  | 0.1669 | 0.1118 |
|  | 0.1455 | 0.1006 |

N.D., no decision
