# Supplementary material for: Cathodal tDCS exerts neuroprotective effect in rat brain after acute ischemic stroke
Source: BMC Neurosci. 2020 May 12;21:21. doi: 10.1186/s12868-020-00570-8 (PMC7216334; doi:10.1186/s12868-020-00570-8)
Supplement: Supplementary file 4 — Additional file 4: Table S4. Protein level of NSE (pg/ml). [file 12868_2020_570_MOESM4_ESM.docx]

**Additional file 4.** Protein level of NSE (pg/ml).

| **Groups** | **NSE** |
| --- | --- |
| **Control + Sham  (n = 3)** | 36.451 |
|  | 53.830 |
|  | 61.252 |
| **Control + tDCS  (n = 3)** | 53.488 |
|  | 48.894 |
|  | 55.468 |
| **MCAO + Sham  (n = 3)** | 377.313 |
|  | 390.279 |
|  | 561.884 |
| **MCAO + tDCS  (n = 3)** | 200.116 |
|  | 235.383 |
|  | 117.678 |
