# Supplementary material for: Cathodal tDCS exerts neuroprotective effect in rat brain after acute ischemic stroke
Source: BMC Neurosci. 2020 May 12;21:21. doi: 10.1186/s12868-020-00570-8 (PMC7216334; doi:10.1186/s12868-020-00570-8)
Supplement: Supplementary file 5 — Additional file 5: Table S5. The number of Nissl bodies. [file 12868_2020_570_MOESM5_ESM.docx]

**Additional file 5.** The number of Nissl bodies.

| **Groups** | **Number of Nissl bodies** |
| --- | --- |
| **Control + Sham  (n = 5)** | 456 |
|  | 452 |
|  | 467 |
|  | 473 |
|  | 448 |
| **Control + tDCS  (n = 5)** | 502 |
|  | 463 |
|  | 470 |
|  | 474 |
|  | 451 |
| **MCAO + Sham  (n = 5)** | 175 |
|  | 150 |
|  | 191 |
|  | 163 |
|  | 152 |
| **MCAO + tDCS  (n = 5)** | 329 |
|  | 326 |
|  | 341 |
|  | 317 |
|  | 336 |
