# Supplementary material for: Cathodal tDCS exerts neuroprotective effect in rat brain after acute ischemic stroke
Source: BMC Neurosci. 2020 May 12;21:21. doi: 10.1186/s12868-020-00570-8 (PMC7216334; doi:10.1186/s12868-020-00570-8)
Supplement: Supplementary file 6 — Additional file 6: Table S6. The percentage of apoptotic cells. [file 12868_2020_570_MOESM6_ESM.docx]

**Additional file 6.** The percentage of apoptotic cells.

| **Groups** | **The percentage of apoptotic cells** |
| --- | --- |
| **Control + Sham  (n = 5)** | 0.00 |
|  | 0.01 |
|  | 0.00 |
|  | 0.01 |
|  | 0.00 |
| **Control + tDCS  (n = 5)** | 0.01 |
|  | 0.00 |
|  | 0.00 |
|  | 0.00 |
|  | 0.01 |
| **MCAO + Sham  (n = 5)** | 0.37 |
|  | 0.52 |
|  | 0.32 |
|  | 0.49 |
|  | 0.63 |
| **MCAO + tDCS  (n = 5)** | 0.15 |
|  | 0.19 |
|  | 0.28 |
|  | 0.21 |
|  | 0.13 |
