# Supplementary material for: Cathodal tDCS exerts neuroprotective effect in rat brain after acute ischemic stroke
Source: BMC Neurosci. 2020 May 12;21:21. doi: 10.1186/s12868-020-00570-8 (PMC7216334; doi:10.1186/s12868-020-00570-8)
Supplement: Supplementary file 7 — Additional file 7: Table S7. The level of apoptosis related protein. [file 12868_2020_570_MOESM7_ESM.docx]

**Additional file 7.** The level of apoptosis related protein.

| **Groups** | **Grey value of Caspase 3** | **Grey value of β-actin** | **Relative expression** |
| --- | --- | --- | --- |
| **Control + Sham  (n = 3)** | 61407 | 321239 | 0.19115674 |
|  | 79983 | 364701 | 0.21931116 |
|  | 61218 | 322139 | 0.19003598 |
| **Control + tDCS  (n = 3)** | 73561 | 366381 | 0.20077733 |
|  | 97346 | 411133 | 0.23677496 |
|  | 63832 | 358359 | 0.17812306 |
| **MCAO + Sham  (n = 3)** | 217582 | 425816 | 0.51097657 |
|  | 261096 | 500146 | 0.52203956 |
|  | 216878 | 447084 | 0.48509452 |
| **MCAO + tDCS  (n = 3)** | 111212 | 441080 | 0.25213567 |
|  | 155369 | 517296 | 0.30034835 |
|  | 112222 | 442632 | 0.25353341 |

| **Groups** | **Grey value of Bcl2** | **Grey value of Bax** | **The ratio of Bcl2/Bax** |
| --- | --- | --- | --- |
| **Control + Sham  (n = 3)** | 321836 | 132630 | 2.42657016 |
|  | 363675 | 127006 | 2.8634474 |
|  | 354307 | 119206 | 2.97222455 |
| **Control + tDCS  (n = 3)** | 344742 | 125239 | 2.75267289 |
|  | 365619 | 118003 | 3.09838733 |
|  | 359582 | 111122 | 3.23592088 |
| **MCAO + Sham  (n = 3)** | 441973 | 312879 | 1.4126004 |
|  | 419435 | 311419 | 1.34685103 |
|  | 442587 | 298872 | 1.48085803 |
| **MCAO + tDCS  (n = 3)** | 571179 | 260174 | 2.1953731 |
|  | 551343 | 239588 | 2.30121292 |
|  | 568645 | 262157 | 2.16910096 |
