# Supplementary material for: Cathodal tDCS exerts neuroprotective effect in rat brain after acute ischemic stroke
Source: BMC Neurosci. 2020 May 12;21:21. doi: 10.1186/s12868-020-00570-8 (PMC7216334; doi:10.1186/s12868-020-00570-8)
Supplement: Supplementary file 8 — Additional file 8: Figure S1. Related western blot data in triplicate. [file 12868_2020_570_MOESM8_ESM.docx]

**Additional file 8.** Related western blot data in triplicate.
