# Supplementary material for: Cathodal tDCS exerts neuroprotective effect in rat brain after acute ischemic stroke
Source: BMC Neurosci. 2020 May 12;21:21. doi: 10.1186/s12868-020-00570-8 (PMC7216334; doi:10.1186/s12868-020-00570-8)
Supplement: Supplementary file 9 — Additional file 9: Table S8. The number of GFAP+ and IBA-1+ cells. [file 12868_2020_570_MOESM9_ESM.docx]

**Additional file 9.** The number of GFAP^+^ and IBA-1^+^ cells.

| **Groups** | **The number of GFAP^+^ cells** | **The number of Iba1^+^ cells** |
| --- | --- | --- |
| **Control + Sham  (n = 3)** | 13 | 7 |
|  | 12 | 9 |
|  | 12 | 10 |
| **Control + tDCS  (n = 3)** | 10 | 8 |
|  | 14 | 9 |
|  | 12 | 11 |
| **MCAO + Sham  (n = 3)** | 20 | 20 |
|  | 22 | 23 |
|  | 19 | 25 |
| **MCAO + tDCS  (n = 3)** | 16 | 13 |
|  | 15 | 14 |
|  | 17 | 12 |
