# Supplementary material for: Cathodal tDCS exerts neuroprotective effect in rat brain after acute ischemic stroke
Source: BMC Neurosci. 2020 May 12;21:21. doi: 10.1186/s12868-020-00570-8 (PMC7216334; doi:10.1186/s12868-020-00570-8)
Supplement: Supplementary file 10 — Additional file 10: Table S9. The size of GFAP+ and IBA-1+ cells. [file 12868_2020_570_MOESM10_ESM.docx]

**Additional file 10.** The size of GFAP^+^ and IBA-1^+^ cells.

| **Groups** | **The size of GFAP^+^ cells** | **The size of Iba1^+^ cells** |
| --- | --- | --- |
| **Control + Sham  (n = 3)** | 312.31 | 284.39 |
|  | 366.84 | 256.21 |
|  | 448.17 | 205.43 |
| **Control + tDCS  (n = 3)** | 361.02 | 208.01 |
|  | 395.93 | 276.01 |
|  | 484.42 | 297.65 |
| **MCAO + Sham  (n = 3)** | 3100.66 | 1827.23 |
|  | 4801.77 | 2283.17 |
|  | 3556.26 | 1671.31 |
| **MCAO + tDCS  (n = 3)** | 2701.57 | 1057.14 |
|  | 1724.00 | 1001.31 |
|  | 2268.61 | 734.19 |
