# Supplementary material for: Cathodal tDCS exerts neuroprotective effect in rat brain after acute ischemic stroke
Source: BMC Neurosci. 2020 May 12;21:21. doi: 10.1186/s12868-020-00570-8 (PMC7216334; doi:10.1186/s12868-020-00570-8)
Supplement: Supplementary file 11 — Additional file 11: Table S10. The protein level of GFAP and Iba1. [file 12868_2020_570_MOESM11_ESM.docx]

**Additional file 11.** The protein level of GFAP and Iba1.

| **Groups** | **Grey value of GFAP** | **Grey value of β-actin** | **Relative expression** |
| --- | --- | --- | --- |
| **Control + Sham  (n = 3)** | 92411 | 549959 | 0.16803253 |
|  | 112205 | 613041 | 0.18303017 |
|  | 81127 | 474996 | 0.17079512 |
| **Control + tDCS  (n = 3)** | 125626 | 599891 | 0.20941471 |
|  | 152857 | 677262 | 0.22569847 |
|  | 135357 | 448987 | 0.30147198 |
| **MCAO + Sham  (n = 3)** | 297456 | 587259 | 0.50651586 |
|  | 353678 | 665923 | 0.53110945 |
|  | 273542 | 523003 | 0.52302186 |
| **MCAO + tDCS  (n = 3)** | 154320 | 522612 | 0.29528599 |
|  | 204238 | 585936 | 0.34856708 |
|  | 159906 | 429379 | 0.37241225 |

| **Groups** | **Grey value of Iba1** | **Grey value of β-actin** | **Relative expression** |
| --- | --- | --- | --- |
| **Control + Sham  (n = 3)** | 141665 | 549959 | 0.25759193 |
|  | 108657 | 474996 | 0.22875351 |
|  | 165469 | 613041 | 0.26991506 |
| **Control + tDCS  (n = 3)** | 154860 | 599891 | 0.2581469 |
|  | 127001 | 448987 | 0.2828612 |
|  | 167041 | 677262 | 0.24664162 |
| **MCAO + Sham  (n = 3)** | 273651 | 587259 | 0.46598009 |
|  | 216716 | 523003 | 0.41436856 |
|  | 321986 | 665923 | 0.48351836 |
| **MCAO + tDCS  (n = 3)** | 152240 | 522612 | 0.29130598 |
|  | 108864 | 429379 | 0.25353825 |
|  | 158639 | 585936 | 0.27074459 |
