# Supplementary material for: Cathodal tDCS exerts neuroprotective effect in rat brain after acute ischemic stroke
Source: BMC Neurosci. 2020 May 12;21:21. doi: 10.1186/s12868-020-00570-8 (PMC7216334; doi:10.1186/s12868-020-00570-8)
Supplement: Supplementary file 12 — Additional file 12: Table S11. The level of inflammatory factors. [file 12868_2020_570_MOESM12_ESM.docx]

**Additional file 12.** The level of inflammatory factors.

| **Groups** | **IL-6** | **IL-1b** | **TNF-a** | **IL-10** |
| --- | --- | --- | --- | --- |
| **Control + Sham  (n = 3)** | 373.568 | 175.002 | 181.76 | 187.680 |
|  | 316.875 | 204.312 | 185.93 | 190.108 |
|  | 380.110 | 201.764 | 198.43 | 182.130 |
| **Control + tDCS  (n = 3)** | 399.734 | 189.748 | 219.27 | 193.923 |
|  | 409.546 | 199.579 | 210.94 | 187.680 |
|  | 314.695 | 196.302 | 195.93 | 190.454 |
| **MCAO + Sham  (n = 3)** | 826.035 | 394.789 | 1184.37 | 302.901 |
|  | 815.338 | 402.799 | 1208.40 | 397.590 |
|  | 735.525 | 374.399 | 1123.20 | 322.671 |
| **MCAO + tDCS  (n = 3)** | 383.183 | 312.502 | 312.50 | 575.526 |
|  | 509.25 | 336.533 | 336.53 | 583.851 |
|  | 593.295 | 331.435 | 331.44 | 702.471 |
