# Supplementary material for: Cathodal tDCS exerts neuroprotective effect in rat brain after acute ischemic stroke
Source: BMC Neurosci. 2020 May 12;21:21. doi: 10.1186/s12868-020-00570-8 (PMC7216334; doi:10.1186/s12868-020-00570-8)
Supplement: Supplementary file 13 — Additional file 13: Table S12. Animal distribution. [file 12868_2020_570_MOESM13_ESM.docx]

**Additional file 13.** Animal distribution.

| **Groups** | **Body weight;**  **mNSS Score;** | **TTC staining** | **ELISA of NSE, IL-6, IL-1β, TNF-α and IL-10** | **Frozen section staining of GFAP and IBA-1** | **Western blot of GFAP, IBA-1, Caspase-3, Bcl-2, Bax and β-actin** | **Paraffin section staining of Nissl bodies, HE and TUNEL** |
| --- | --- | --- | --- | --- | --- | --- |
| **Control + Sham**  **(n = 27)** | 6 | 3 | 5 | 3 | 5 | 5 |
| **Control + tDCS**  **(n = 27)** | 6 | 3 | 5 | 3 | 5 | 5 |
| **MCAO + Sham**  **(n = 27)** | 6 | 3 | 5 | 3 | 5 | 5 |
| **MCAO + tDCS**  **(n = 28)** | 7 | 3 | 5 | 3 | 5 | 5 |
